# Scalable extraction of pongamol from *Pongamia pinnata* for synthesis and application of colloidally stable iron nanoparticles

**DOI:** 10.1039/d5ra04181c

**Published:** 2025-12-08

**Authors:** Renjie Cui, Mengying Wu, Anna Galang, Franklin Perez, Vanessa Partrige Hing, Nesha May Octavio Andoy, Ruby May Arana Sullan, Kris Sanghyun Kim

**Affiliations:** a Department of Physical and Environmental Sciences, University of Toronto Scarborough 1065 Military Trail Toronto ON M1C 1A4 Canada kris.kim@utoronto.ca ruby.sullan@utoronto.ca; b Department of Chemistry, University of Toronto 80 St. George St. Toronto ON M5S 3H6 Canada

## Abstract

Pongamol, a flavonoid that constitutes a major bioactive fraction of *Pongamia pinnata* oil, is attracting interest as a renewable antioxidant, antimicrobial and anti-inflammatory agent. Its wider use is hindered by labor-intensive schemes that rely on preparative chromatography. Here we introduce a scalable two-solvent recrystallization (ethanol : water) that yields pongamol at high purity (>97%), eliminating the need for more sophisticated instrumentation. Extraction at pH 5.8 followed by the recrystallization achieved high recovery while suppressing co-extractables. UV-Vis, ^1^H/^13^C-NMR, LC-MS and HPLC verified the identity and isomeric integrity of the product. We further show that purified pongamol serves simultaneously as a reducing agent and capping ligand for a one-pot, room-temperature “green” synthesis of iron nanoparticles (FeNPs). The resulting FeNPs exhibited a mostly spherical morphology, a mean hydrodynamic diameter of ∼12 nm, and a negative zeta potential that conferred colloidal stability comparable to, or better than, FeNPs synthesized with green tea extract, as confirmed by DLS and TEM. This dual-purpose protocol integrates bio-based extraction with benign nanoparticle synthesis, offering an efficient route to value-added utilization of *P. pinnata* oil in sustainable nanomaterials. Moreover, we also demonstrated synthesized pongamol-FeNPs can degrade methylene blue effectively as a photocatalyst and promote *Pseudomonas defensor* growth at low concentrations.

## Abstract 2

Pongamol, a flavonoid from *Pongamia pinnata*, was purified through two-solvent recrystallization and applied towards the synthesis of pongamol-FeNPs which were further tested for bacterial viability with *P. defensor* and methylene blue dye degradation.

## Graphical abstract

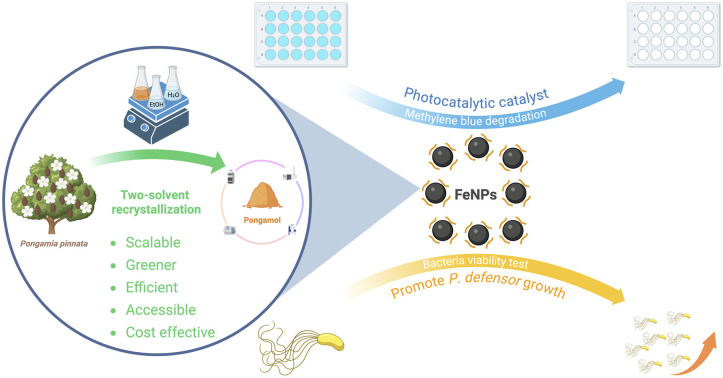

## Introduction

**Fig. 1:**
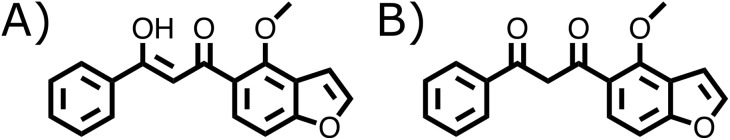
Keto–enol tautomers of pongamol observed during extraction and purification. (A) Pongamol structure containing one hydroxyl group and one carbonyl group. (B) Pongamol structure containing two carbonyl groups. Chemical structures were drawn using ChemDraw version 20.1.0.112.

*Pongamia pinnata*, commonly known as Karanja, is a leguminous tree native to India and Southeast Asia whose seeds contain 27–39% oil, 20–30% protein, and a variety of flavonoids.^1,2^ Among these, pongamol (C_18_H_14_O_4_, Fig. 1), a polyphenolic chalcone compound, has been shown to confer antioxidant, antimicrobial, anti-inflammatory, anti-diabetic and anti-hyperglycemic activities.^3–8^ Due to its wide range of potential health benefits, there has been an upsurge of research focusing on the properties, activities, and potential applications of pongamol and other compounds found in Karanja oil. Beyond pharmacological applications, *Pongamia pinnata* seed, or leaf, extracts have also gained popularity in the synthesis of nanoparticles due to the ability of flavonoids to act as both a reducing and stabilizing agents for the green synthesis of metal nanoparticles.^6,7^ Pongamol, in particular, contains hydroxyl and carbonyl groups that enable it to act both as a reducing agent to synthesize nanoparticles and as a capping agent to further stabilize the nanoparticle through its high affinity and adsorption properties toward metal ions.^8–12^ Thus, pongamol could potentially be used to synthesize metal nanoparticles.

However, the methods for extracting and purifying pongamol from Karanja oil are still quite limited, often requiring the use of organic solvents, followed by acid hydrolysis to obtain crude pongamol.^5,13,14^ This is frequently followed by purification involving silica gel chromatography for separation then thin layer chromatography (TLC) for confirmation, or high performance liquid chromatography (HPLC) while using petroleum ether, chloroform and ethyl acetate as solvents and mobile phase.^2,15,16^ These workflows are solvent-intensive, time-consuming and require extra instruments or apparatus like silica gel columns with TLC and HPLC, limiting scalability.

Recrystallization offers a simpler alternative. In particular, two-solvent recrystallization (Scheme S1) involves dissolving the crude product in a good solvent (solvent A), followed by a controlled addition of a second solvent (solvent B) that is miscible with solvent A. This continues until the solution turns cloudy, indicating the onset of precipitation. A small amount of solvent A is again introduced until the solution turns clear. Upon cooling, the purified product crystallizes out and is collected by vacuum filtration. This approach eliminates the need for column chromatography, offering a simpler and more scalable alternative for purification.

In parallel, there has been a growing interest in sustainable and environmentally benign approaches for synthesizing nanoparticles, often referred to as “green synthesis.” These methods aim to minimize the use of hazardous chemicals and energy-intensive processes by leveraging natural reducing and stabilizing agents derived from plant extracts, microorganisms, or biowaste. Such approaches not only reduce ecological and health risks associated with conventional synthesis routes but also offer cost-effective and scalable alternatives, aligning with the principles of green chemistry.^17–19^ In this context, bioactive compounds like pongamol—readily available from renewable sources such as *Pongamia pinnata*—are of particular interest due to their dual function as both reducing and capping agents in nanoparticle formation.

**Scheme 1:**
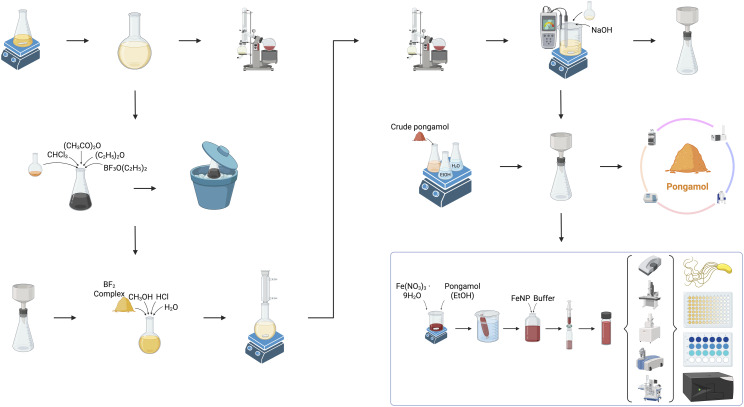
Schematic overview of the extraction, purification, and application of pongamol from *Pongamia pinnata* oil. The procedure begins with solvent extraction of pongamol from Karanja oil, followed by acid hydrolysis and removal of BF_2_ complexes. Crude pongamol is then purified *via* two-solvent recrystallization using ethanol and water, eliminating the need for chromatographic separation. The purified pongamol is characterized by ultraviolet-visible spectrophotometer (UV-Vis), nuclear magnetic resonance (NMR), liquid chromatography and mass spectrometry (LC-MS), and high performance liquid chromatography (HPLC). In the final step, pongamol is used as both a reducing and capping agent in the synthesis of iron nanoparticles (FeNPs) from Fe(NO_3_)_3_·9H_2_O in ethanol. The synthesized FeNPs are stabilized in buffer and characterized using dynamic light scattering (DLS), phase analysis light scattering (PALS), scanning electron microscope – energy dispersive X-ray spectroscopy (SEM-EDS), transmission electron microscopy (TEM), attenuated total reflectance-fourier transform infrared (ATR-FTIR) and X-ray photoelectron spectroscopy (XPS). Synthesized pongamol-FeNPs were also applied for bacteria viability tests with *Pseudomonas defensor* and dye degradation with methylene blue.

Here we (i) develop a scalable ethanol–water two-solvent recrystallization (Scheme 1) that yields pongamol at high purity and (ii) demonstrate its dual role as reducing and capping agent in the green synthesis of iron nanoparticles (FeNPs). We also explore the application of resulting pongamol-FeNPs towards the degradation of methylene blue and test for bacterial viability with *P. defensor*.

## Experimental

### Pongamol isolation from Karanja oil

Karanja seeds oil (50 g, Botanic Plant) was first extracted with methanol (100 mL) (Sigma-Aldrich, HPLC grade) for 24 h and repeated 3× followed by evaporation of solvent until around 25 mL volume remained (Scheme 1). All concentrated methanol solution was then treated successively with chloroform (CHCl_3_) (Sigma-Aldrich), acetic anhydride (CH_3_CO)_2_O(Sigma-Aldrich), diethyl ether (C_2_H_5_)_2_O(Sigma-Aldrich), and boron trifluoride diethyl etherate (BF_3_O(C_2_H_5_)_2_) (Sigma-Aldrich) with a ratio of 5 : 1 : 2 : 3 : 2, respectively, then placed in an ice bath for 48 h. During this time, pongamol complex was formed in the solution as yellow crystals. To recover the pongamol BF_2_ complex, the solutions were vacuum filtered and air-dried. Pongamol BF_2_ complex was dissolved in methanol and 6 M hydrochloric acid (HCl) (Sigma-Aldrich) at a 9 : 1 (v/v) ratio, which was then hydrolyzed at 70 °C for 6 h. Next, the solution was concentrated by evaporating the methanol then adjusted the pH to around 5.8 with sodium hydroxide (NaOH) (ACP). The crude pongamol was collected after vacuum filtration.

### Purification of pongamol

Two-solvent recrystallization with ethanol and water was used for the purification of the pongamol. Crude pongamol was dissolved into minimum amount of hot ethanol, after which Milli-Q water (18 MΩ cm) was slowly added with swirling until the solution remained cloudy. Hot ethanol was then further added drop by drop until the solution turned clear. The solution was left cool to room temperature, after which it was placed into the ice bath to allow for crystal formation. The resulting precipitate was collected by vacuum filtration.

### Characterization and quantification

#### Ultraviolet-visible spectrophotometer (UV-Vis)

Pongamol was prepared with concentration of 10 µg mL^−1^ and quantified in the UV region (350 nm) using a Cary 60 UV-vis spectrophotometer (Agilent Technologies). Standard pongamol solutions were prepared at concentrations of 1 µg mL^−1^, 2 µg mL^−1^, 5 µg mL^−1^, 10 µg mL^−1^ and 20 µg mL^−1^.

#### Nuclear magnetic resonance (NMR) spectroscopy

Recrystallized pongamol samples (20 mg) were dissolved in 1.0 mL of deuterated chloroform for separate ^1^H NMR and ^13^C NMR analyses, which were recorded at 500 MHz on an Ultra Shield 500 MHz instrument (Bruker). MNOVA was used to analyze the ^1^H and ^13^C NMR spectra. ^1^H and ^13^C NMR spectral chemical shifts and coupling constants were expressed in *δ* and Hz respectively.

#### High performance liquid chromatography (HPLC)

Methanol (Sigma-Aldrich, HPLC grade) was used as the blank, and pongamol (Toronto Research Chemicals (TRC), purity 97.8%) was used as the standard for generating a calibration curve with concentrations ranging from 1 µg mL^−1^ to 20 µg mL^−1^. Spiked and non-spiked samples at 10 µg mL^−1^ were prepared with and without the standard in the isolated pongamol, respectively. All samples were filtered through a 0.22 µm syringe filter (Fisher Scientific). Reverse phase HPLC analysis was carried out with a 1200 Infinity System (Agilent) and Eclipse XBD-C18 column (ZORBAX; 4.6 mm × 150 mm, 5 µm particle size), with pump (Agilent 1200). A 20 µL injection volume was used with an autosampler (Agilent 1100), and chromatograms were monitored using a variable-wavelength detector (VWD, Agilent 1200) at *λ* = 350 nm and *λ* = 300 nm. An isocratic elution method was employed, using a mobile phase containing a mixture of methanol, water, and acetic acid (ACP) in a ratio of 85 : 13.5 : 1.5, filtered through a 0.22 µm nylon filter prior to use.^14^ The flow rate was set to 0.5 mL min^−1^, and the run time for each sample was 30 min. The purity and relative percentage of pongamol were quantified based on the peak area.

#### Liquid chromatography and mass spectrometry (LC-MS)

LC-MS was carried out on a 6530 accurate-mass quadrupole time-of-flight (Q-TOF, Agilent). Electrospray ionization (ESI) with positive ionization mode was utilized to characterize pongamol and karanjin.

### Iron nanoparticle (FeNP) synthesis and characterization

Pongamol was prepared in anhydrous ethanol (commercial alcohols) at 100 µg mL^−1^, 250 µg mL^−1^, 500 µg mL^−1^, 750 µg mL^−1^, 800 µg mL^−1^, 900 µg mL^−1^, 1000 µg mL^−1^ and 2000 µg mL^−1^. 2 M iron(iii) nitrate nonahydrate (Fe(NO_3_)_3_·9H_2_O, Sigma-Aldrich) was prepared in 1 mM pH 4 sodium acetate buffer (Sigma-Aldrich) adjusted with acetic acid (ACP). FeNPs were synthesized by slowly adding 1.5 mL of pongamol ethanol solution in increments of 100 µL into 4.5 mL of 2 M Fe(NO_3_)_3_·9H_2_O while stirring. The mixture was continuously stirred for five minutes after the addition of pongamol was completed. FeNP solutions were dialyzed under a pH of 4, comprised of sodium acetate buffer, and the dialysis bath was refreshed three times over a 24 hours period. The FeNP solutions were then collected and diluted with 1 mM sodium acetate buffer at 1 : 1 ratio and further filtered through a 0.22 µm nylon filter.

Green tea extract was prepared in MilliQ-water after heating the green tea for 20 min at around 80 °C and filtration (Fisher brand). FeNPs were synthesized by slowly adding 4 mL of 4000 µg mL^−1^ green tea extract in increments of 100 µL into 5 mL of 2 M Fe(NO_3_)_3_·9H_2_O while stirring. The mixture was continuously stirred for five minutes after the addition of green tea was completed. FeNP solutions were dialyzed under a pH of 4, comprised of sodium acetate buffer, and the dialysis bath was refreshed three times over a 24 hours period. The FeNP solutions were then collected and diluted with 1 mM sodium acetate buffer at 1 : 1 ratio and further filtered through a 0.22 µm nylon filter.

The size of the FeNPs were monitored using dynamic light scattering (DLS, NanoBrook Omni, Brookhaven Instruments) and surface zeta potential was measured using phase analysis light scattering (PALS, NanoBrook Omni, Brookhaven Instruments). FeNPs were diluted 1 : 1 with sodium acetate buffer for DLS measurements, while a 1 : 3 dilution was used for surface zeta potential measurements. Transmission electron microscopy (TEM, Hitachi H7500) was performed to visualize the FeNPs. Synthesized FeNPs were centrifuged (Beckman Coulter, Avanti J-30I) at 108 8000 G for 25 min. The settled particles were collected and washed with water for three times, then resuspended in sodium acetate buffer. 20 µL of diluted FeNPs was drop-casted onto Cu-coated TEM grid (Electron Microscopy Science, CF300-CU-50). Pongamol-FeNPs was also added onto the surface of carbon tape with air dry. Scanning electron microscope – energy dispersive spectroscopy (SEM-EDS) was performed using Zeiss 360 VP (Oxford). The collected FeNPs after centrifuging were also collected and resuspended in water, subsequently freeze-dried (Harvest Right) for attenuated total reflectance-fourier transform infrared (ATR-FTIR, Bruker) and X-ray photoelectron spectroscopy (XPS) analysis.

XPS analysis was conducted using a K-Alpha XPS system (ThermoFisher Scientific, East Grinstead (UK)). X-rays were monochromatized Al K-Alpha x-rays, with a 400 µm spot size (2 : 1 ellipse, with the major axis being the noted spot size). A low-energy electron/ion (Ar) flood source was used for charge neutralization. Survey spectra were acquired at a pass energy of 200 eV. The corresponding point density on the energy axis was 1 eV per step. These scans were performed to identify the species detectable on the surface. Regional scans were performed at 50 eV pass energy, and with a point density of 0.1 eV between points. The dwell time was 50 ms.

### Kinetic degradation test of FeNPs as a Fenton reaction catalyst

The steady-state kinetics assay was performed at room temperature in a 24-well microplate (*n* = 3). Each well contained 500 µL of methylene blue (MB, Sigma) prepared in 1 mM pH 4 sodium acetate buffer at varying concentrations (0.1, 0.075, 0.05, 0.025, 0.01, 0.0075, 0.005, 0.0025, and 0.001 mM), 0.13 mg of pongamol-FeNPs, and 100 µL of 30% hydrogen peroxide (H_2_O_2_, Sigma). The reactions were monitored every minute at 664 nm using a microplate reader (Infinite® 200 Pro, TECAN). MB concentrations were determined from absorbance values using a calibration curve from 0.1 mM to 0.001 mM, and the initial degradation rate was calculated based on the slope of the concentration values at 0, 1, and 2 minutes.

### Bacterial viability test of *Pseudomonas defensor*

#### Preparation of *P. defensor* liquid culture

The bacterial strain *P. defensor* was cultured in a Falcon tube containing LB medium and incubated in static condition at 27 °C overnight. Following incubation, the bacterial culture was centrifuged at 6000 rpm for 5 minutes using an Eppendorf 5804/5804 R Benchtop Centrifuge. The supernatant was discarded, and the bacterial pellet was resuspended in 1× PBS. The centrifugation and resuspension steps were repeated once more to ensure thorough washing of the bacteria. The final bacterial suspension was then resuspended in 0.85% (w/v) NaCl solution. To determine bacterial concentration, the optical density at 600 nm (OD_600_) was measured using the IMPLEN OD600 DiluPhotometer™ (product code: IMP OD600-10) with a calibrated blank of 0.85% w/v NaCl solution.

#### Iron nanoparticle coating onto *P. defensor*

Five experimental conditions were prepared in separate tubes: 0.85% w/v NaCl as a control and 0.85% w/v NaCl with FeNP solution at 0.01, 0.1, 1 and 5 mg mL^−1^ concentrations. A bacterial suspension (OD_600_ = 0.01) was added to each condition, and the solutions were incubated at room temperature for 1 hour.

#### Plating the bacteria-FeNP solution on solid media (King's B agar plates)

To evaluate bacterial growth, 10 µL aliquots from each experimental condition were plated onto King's B agar plates. Three replicate plates were prepared for each condition, with 3 different colony-forming unit (CFU) magnitudes generated through serial dilutions to facilitate colony counting. This approach ensures reliable quantification of bacterial growth following coating under different experimental conditions.

#### Assessment on liquid media (planktonic growth)

To assess planktonic growth, 10 µL of the bacteria-FeNP solutions were added to 90 µL of King's B medium in a 96-well plate. Planktonic growth was monitored at 27 °C with shaking for 24 hours using Tecan Infinite® M Nano + dual-mode microplate reader with monochromator optics.

## Results and discussion

### Optimized extraction at pH 5.8 followed by two-solvent recrystallization yields highly pure pongamol

Crude pongamol was initially extracted following the method reported by Rekha *et al.*, in which Karanja seed oil was treated with petroleum ether and methanol, followed by chemical complexation with boron trifluoride to form a BF_2_–pongamol complex.^16^ The product was then hydrolyzed, neutralized, and purified by column chromatography to obtain crystalline pongamol. In this work, the extraction protocol was further optimized by incorporating a pH adjustment step after hydrolysis, inspired and adapted from Rao *et al.*.^20^ The pH adjustment neutralizes the small amount of hydrofluoric acid (HF) produced during the process of hydrolysis and dissociation of boron trifluoride diethyl etherate (BF_3_O(C_2_H_5_)_2_) with water, which can lower the solution pH and slow the hydrolysis rate.^20^ To facilitate more complete hydrolysis, the solution was adjusted to pH 5.8 with addition of NaOH prior to crude pongamol product collection. To further purify the crude pongamol, two-solvent recrystallization was performed using ethanol and water. These solvents were selected as they are miscible with one another, however, amongst the two solvents, ethanol can dissolve crude pongamol while water cannot, which are important parameters to apply two-solvent recrystallization.

**Fig. 2:**
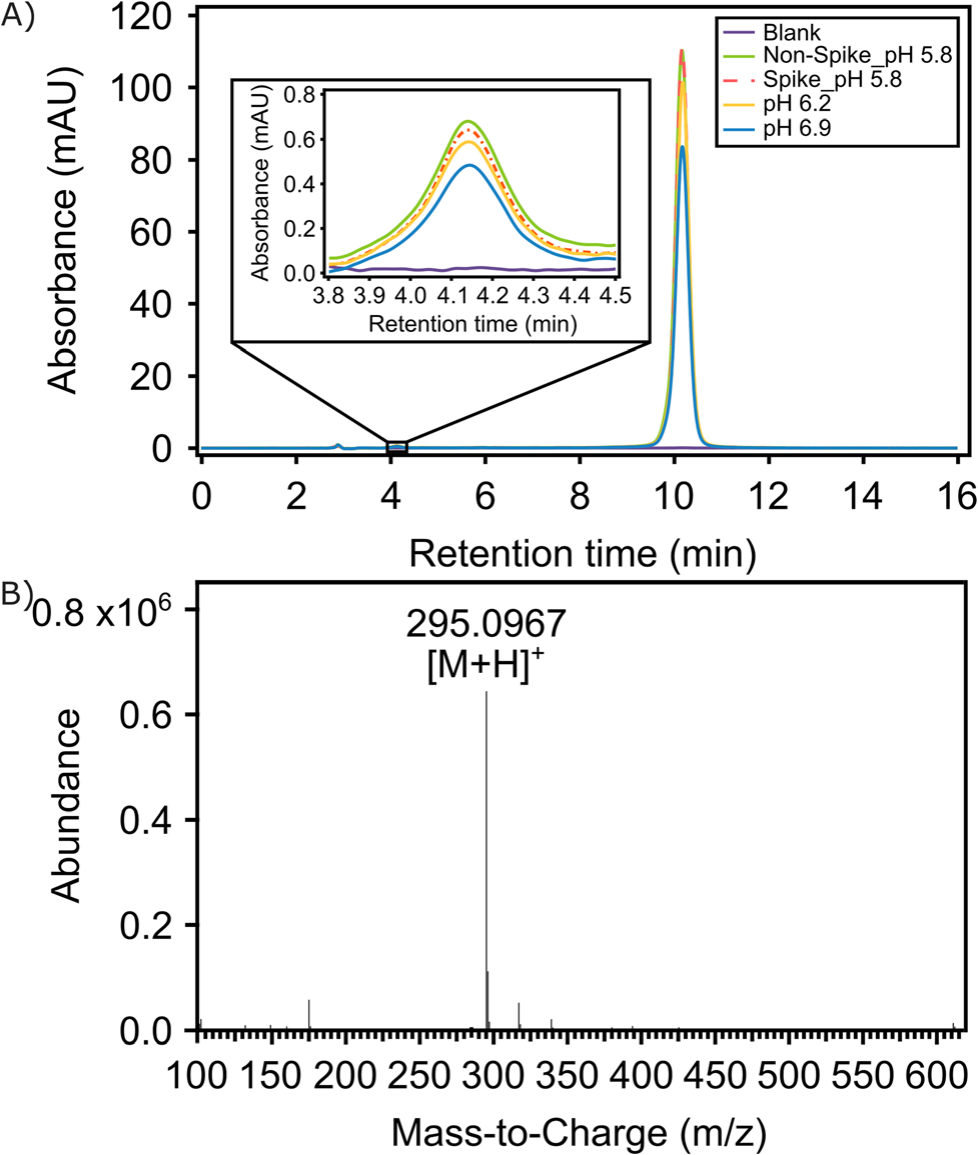
(A) High performance liquid chromatography (HPLC) chromatogram with blank (purple), non-spiked pongamol (green), spiked pongamol (red), pongamol extracted at pH 6.2 (yellow) and pongamol extracted at pH 6.9 (blue) samples. (B) Liquid chromatography and mass spectrometry (LC-MS) spectra of extracted pongamol.

HPLC was applied to quantify the purity of the isolated pongamol obtained with the aforementioned pH adjustment and two-solvent recrystallization. The chromatogram of purified pongamol exhibited a peak at approximately 10 minutes (Fig. 2A). The sample was also spiked with standard pongamol (obtained from Toronto Research Chemicals (TRC)) and the chromatogram showed a peak at the same retention time, indicating the extracted pongamol shares similar properties as the reference material. For quantitative applications, as shown in the HPLC chromatograms of pongamol at 350 nm in Fig. S1, the calibration curve for pongamol exhibited strong linearity between 1 µg mL^−1^ to 20 µg mL^−1^ (*R*^2^ = 1.000).

A small peak at a retention time around 4 min was noted (Fig. 2A). Karanjin has often been found to be a major component of Karanja oil which could be detected at wavelength of 300 nm while pongamol absorbs at 350 nm.^14^ Thus, we suspect this minor peak to arise from karanjin or a different isomer of pongamol and the eluting compound was analyzed at a wavelength of 300 nm to verify whether this peak represented karanjin. As shown in the HPLC results that compare the peak areas at wavelengths of 300 nm and 350 nm, at retention times of 4 min and 10 min (Table S1), the peak area at retention time of 4 min was observed to increase when the wavelength was adjusted from 350 nm to 300 nm. This may imply the presence of karanjin in both the extracted product and the standard, though at significantly lower concentrations relative to pongamol (at 10 min).

Rao *et al.* have reported that performing the pongamol extraction at pH 5.8 resulted in the highest purity (98.2% to 99.6%) of the compound, *versus* pH 7.^20^ For testing the importance and the relationship between pH and the purity of pongamol while exploring the opportunities for further optimization in the pH range between 5.8 and 7.0, the pH of the crude pongamol was also adjusted to 6.2 and 6.9 in our work. As can be seen from the quantitative HPLC peak area data and extraction efficiency of pongamol at different pH levels (Tables S2 and S3), the purity of extracted pongamol was highest (>97%; noting there is a small, though insignificant, presence of possibly karanjin) when the pH was adjusted to 5.8 prior to purification, resulting in 0.18 w/w% of pongamol in Karanja oil. As the pH was increased, the purity of the product was observed to decrease to 78% at pH 6.9, which may be due to the presence of intractable products obtained as the pH approaches neutral conditions of 7.^20^ The extraction was also performed under basic conditions where the pH was adjusted to 10, though this resulted in minimal product upon filtration.

### Pongamol was identified as the major compound and the most abundant component in the isolated and purified product, as confirmed by LC-MS (Liquid chromatography – mass spectrometry)

LC-MS analysis of the purified compound, conducted in positive electrospray ionization (ESI^+^) mode, resulted in a molecular ion peak at *m*/*z* 295.1, corresponding to the [M + H]^+^ species of pongamol. This peak was also the base peak in the spectrum (Fig. 2B), confirming its dominance in the sample. Karanjin, another known constituent of Karanja oil, was detected as a minor peak during HPLC analysis.^2^ To verify its presence, a target ion at *m*/*z* 293, corresponding to [M + H]^+^ of karanjin was analyzed in LC-MS separately (Fig. S2 shows the LC-MS confirmation of karanjin). However, both the target score and counts were substantially lower than those for pongamol, indicating that karanjin was present only in trace amounts, with pongamol being the predominant compound in the purified extract.

### Two pongamol structures were identified in the final product by NMR

**Fig. 3:**
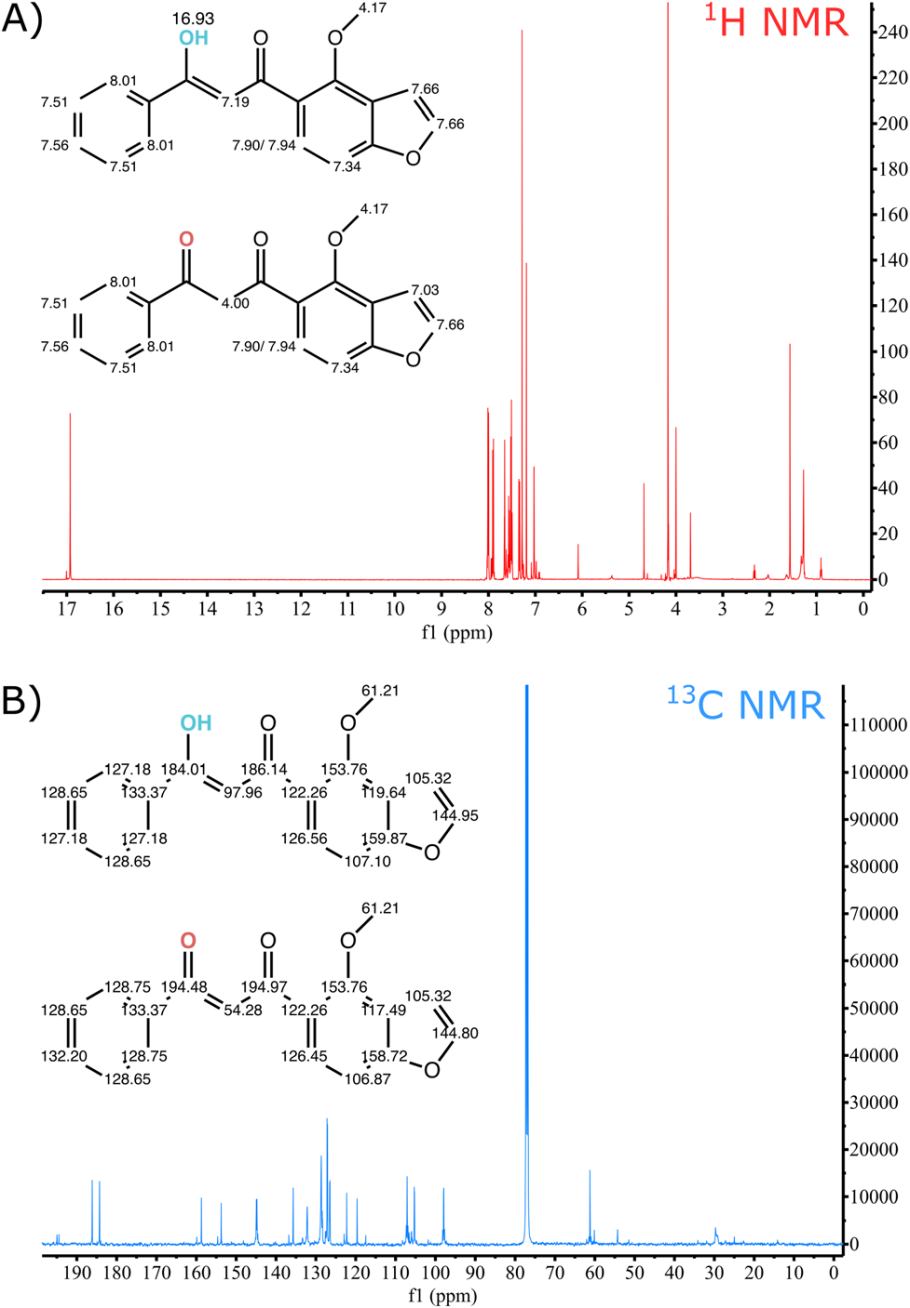
(A) ^1^H nuclear magnetic resonance (NMR) spectra and (B) ^13^C NMR spectra of recrystallized pongamol obtained from *Pongamia pinnata* oil.

To further confirm the identity of the purified compound, samples were analyzed using both ^1^H NMR and ^13^C NMR. According to the ^1^H NMR (Fig. 3A) and ^13^C NMR (Fig. 3B) spectra, along with peak assignments in Table S4, the two-solvent recrystallized product consists of both isomers of pongamol. The spectra display peaks corresponding to the isomer containing one carbonyl and one hydroxyl group (Fig. 1A) as well as the isomer containing two carbonyl groups (Fig. 1B). In addition, majority of the peaks present in both ^1^H and ^13^C NMR can be attributed to the presence of the two pongamol isomers, further highlighting the purity of the isolated product in alignment with the HPLC results.

### Pongamol was quantified with ultraviolet-visible spectroscopy (UV-Vis) as a simpler method and showed similar results with HPLC

**Fig. 4:**
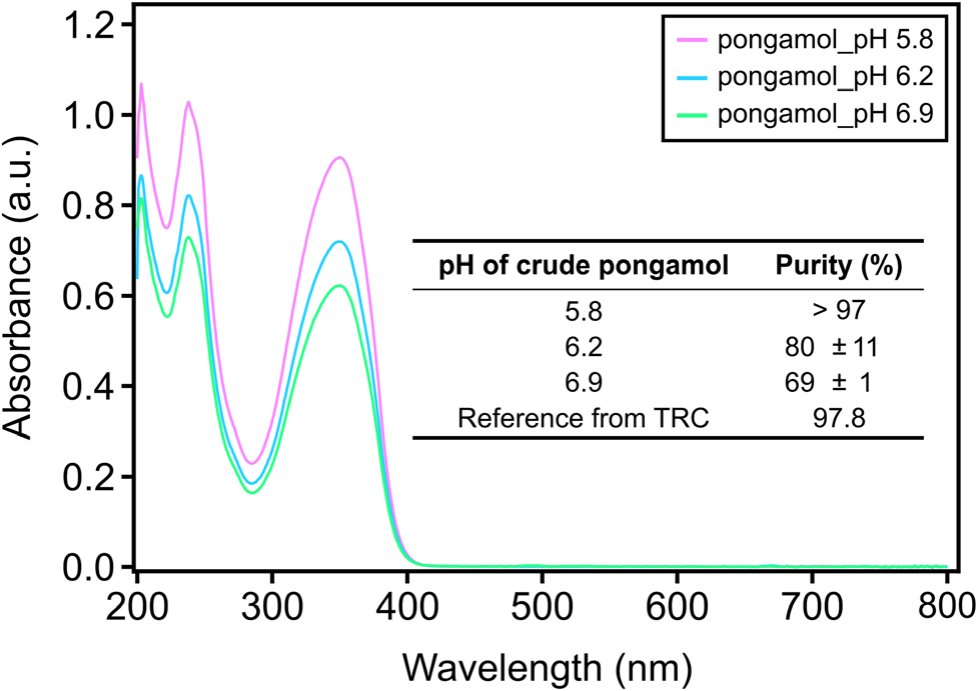
Ultraviolet-visible spectrophotometer (UV-Vis) spectrum of pongamol at pH 5.8 (pink), pH 6.2 (blue), pH 6.9 (green). Insert: table of purity of extracted pongamol as a function of pH determined by UV-Vis (*n* = 3).

Based on the HPLC results, the pongamol extracted after the pH was adjusted to 5.8 offered the highest purity (>97%), while those adjusted to pH 6.2 and 6.9 contain some impurities. However, as seen in the HPLC result, impurities in the pongamol extracted at pH 6.2 and 6.9 were not detected at 350 nm (Fig. 2A). Therefore, UV-Vis was utilized to showcase an alternative method for quantifying pongamol. Strong linearity was observed at concentrations between 1 µg mL^−1^ and 20 µg mL^−1^ with *R*^2^ = 0.999 and the resulting calibration curve was used to quantify extracted pongamol samples (Fig. S3). As shown in Fig. 4, the peak absorbance at 350 nm decreased as the pH of the crude pongamol increased and the purity of pongamol was highest, at around 100%, after the pH was adjusted to 5.8. The purity was observed to decrease to 80% and 69% as the pH increased to 6.2 and 6.9, respectively, which aligned with the HPLC quantification results (Table S3).

### Iron nanoparticles (FeNPs) were synthesized with various concentrations of pongamol and exhibited long-term stability

**Fig. 5:**
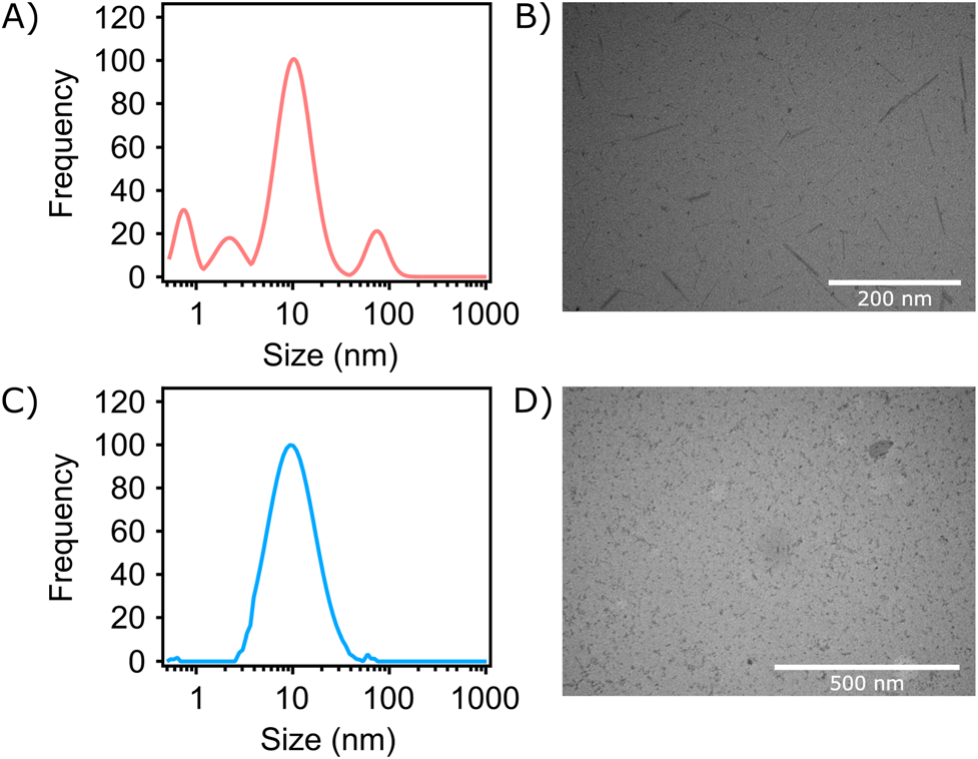
Pongamol-FeNPs were synthesized with 2 M Fe(NO_3_)_3_·9H_2_O and 100 µg mL^−1^ pongamol at 3 : 1 (v/v) ratio. Green tea (GT)-FeNPs were synthesized with 2 M Fe(NO_3_)_3_·9H_2_O and 4000 µg mL^−1^ green tea extract at 5 : 4 (v/v) ratio. (A) Size distribution of pongamol-FeNPs. (B) Transmission electron microscopy (TEM) images of pongamol-FeNPs. (C) Size distribution of GT-FeNPs. (D) TEM images of GT-FeNPs.

In addition to optimizing a method for extracting and purifying pongamol, the resulting product was also used to assess its ability to facilitate the synthesis of FeNPs, offering a potentially green method of synthesis that avoids the need for harsh reducing agents. To evaluate its applicability, a comparative study was conducted with FeNPs synthesized using green tea extract (GT), a well-established natural product which also act as both a reducing and stabilizing agent.^21,22^ The size and surface zeta potential of pongamol-FeNPs synthesized with 2 M Fe(NO_3_)_3_·9H_2_O and 100 µg mL^−1^ pongamol at 3 : 1 (v/v) ratio, as well as GT-FeNPs synthesized with 2 M Fe(NO_3_)_3_·9H_2_O and 4000 µg mL^−1^ green tea extract at 5 : 4 (v/v) ratio were measured using dynamic light scattering (DLS) and phase analysis light scattering (PALS). Results showed the hydrodynamic diameter with the highest frequency for both pongamol-FeNPs and GT-FeNPs was at around 10 nm. The hydrodynamic size distributions of pongamol-FeNPs in solution were observed centered around 1 nm, 2 nm, and 75 nm while GT-FeNPs only showed one main peak at around 10 nm (Fig. 5A and C). From the transmission electron microscopy (TEM) images, the physical size of both dried pongamol-FeNPs and dried GT-FeNPs with spherical shapes were observed, with diameters of around 3 nm (Fig. 5B and D). However, elongated structures with lengths of around 75 nm were also observed in the pongamol-FeNP samples (Fig. 5B). Chalcones, which contain either two carbonyl groups or a hydroxyl and carbonyl group and resemble pongamol, have been reported to act as chelators that coordinate with metal ions to form flavonoid–iron complexes that exhibit elongated shapes.^23^ These undefined elongated structures have previously been observed when nanoparticles were synthesized in an ethanol/methanol mixture environment, similar to the method used in our FeNPs synthesis.^24^ We also suspect that the formation of elongated structures could be influenced by NO_3_^−^ from ferric nitrate, which acts as the counter ion and forms FeNPs with different morphologies, although further analysis is required to confirm the composition of these structures.^25^

**Fig. 6:**
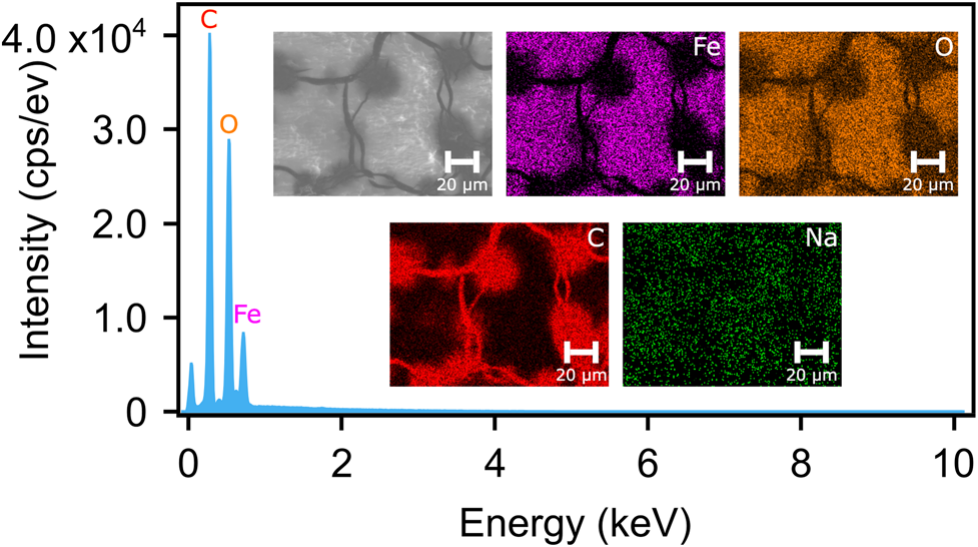
Energy dispersive spectroscopy (EDS) spectrum of pongamol-FeNPs. Insert: scanning electron microscope (SEM) dark-field images and EDS elemental mapping images of Fe, O, C and Na.

The successful synthesis of pongamol-FeNPs and the distribution of Fe and O were further confirmed and investigated using Scanning Electron Microscopy with Energy-Dispersive X-ray Spectroscopy (SEM-EDS). As shown in Fig. 6, EDS mapping of high concentrations of pongamol-FeNPs drop casted on the carbon tape showed high intensity for Fe and O (while no signal was observed for Na) in the pongamol-FeNP regions, which confirmed the formation of FeNPs. However, the pongamol-FeNP region showed a relative lower intensity of *C* at around 0.3 keV. This may be due to the lower amount of *C* in the pongamol-FeNPs relative to the carbon tape, although the edges of the pongamol-FeNPs can still be clearly observed. In addition, according to the EDS spectrum, pongamol-FeNPs showed dominant peaks at around 0.5 keV and 0.7 keV, which correspond to O and Fe in the FeNPs, respectively.

**Fig. 7:**
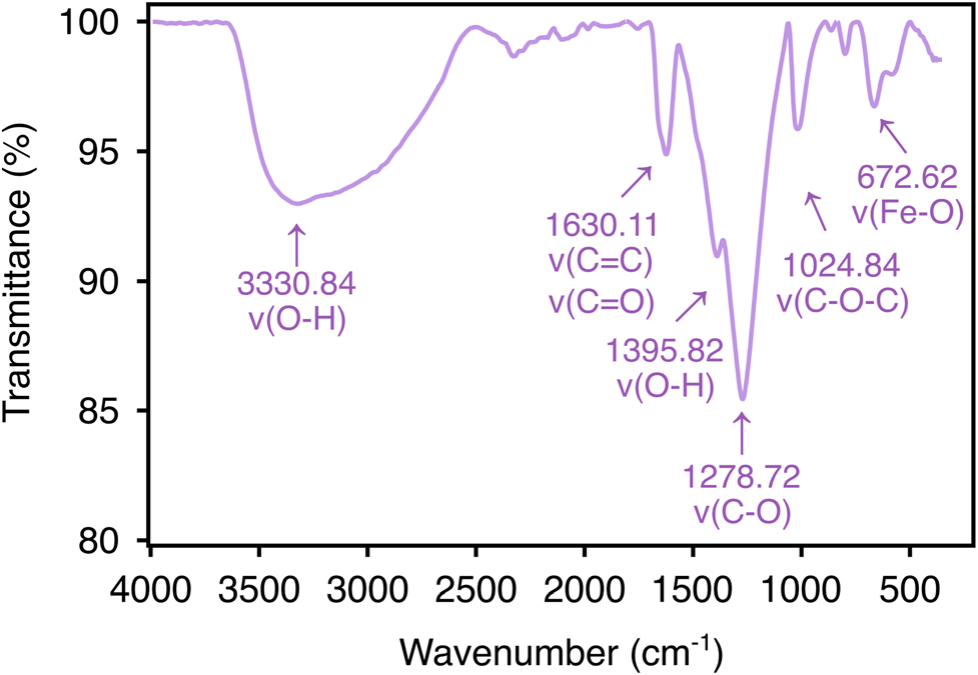
Attenuated total reflectance-fourier transform infrared (ATR-FTIR) spectrum of pongamol-FeNPs.

FTIR was applied to determine the surface composition and the functional groups from pongamol that could act as the reducing and stabilizing agents in synthesizing pongamol-FeNPs. As shown in Fig. 7, both the broad peak at 3330.84 cm^−1^ and the sharp peak at 1395.82 cm^−1^ were assigned to the vibration of O–H bond, which corresponded to the hydroxyl group in pongamol.^26^ The peaks at 1630.11 cm^−1^, 1278.72 cm^−1^ and 1024.82 cm^−1^ could be assigned to C=C or C=O vibration, the C–O bond, and the C–O–C bond, respectively, which were attributed to the carbonyl group from pongamol.^26–29^ Additionally, the peak at 672.62 cm^−1^ can be attributed likely to Fe–O vibration, supporting the potential presence of iron oxide nanoparticles.^30^

**Fig. 8:**
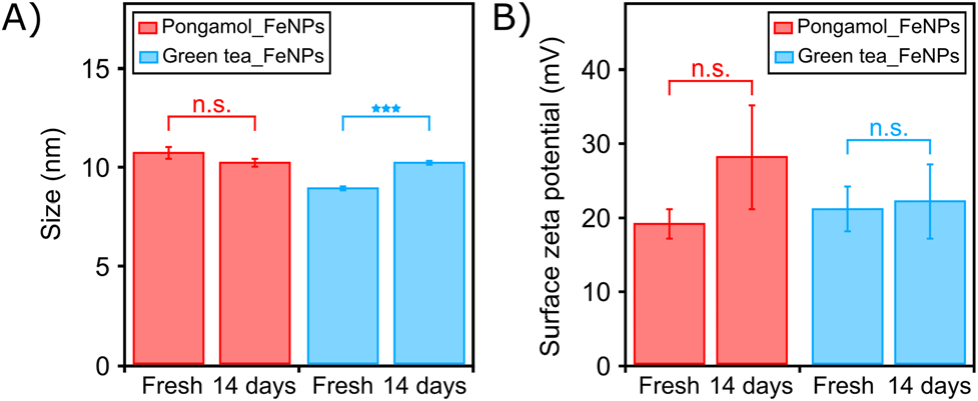
(A) Hydrodynamic diameter and (B) surface zeta potential of pongamol-FeNPs and GT-FeNPs immediately after synthesis and after 14 days (*n* = 3). Statistical analysis was performed with two-way *t*-test. *P* > 0.05 was considered not significant; ***P < 0.001.

The stability of pongamol-FeNPs and GT-FeNPs were assessed through DLS and PALS measurements over a period of 14 days (Fig. 8A and B). The hydrodynamic diameter of pongamol-FeNPs (2 M Fe(NO_3_)_3_·9H_2_O, 100 µg mL^−1^ pongamol, 3 : 1 (v/v) ratio) was measured to be 10.6 ± 0.3 nm when freshly synthesized to 10.2 ± 0.2 nm after 14 days and showed no significant difference, while GT-FeNPs was measured to be 8.9 ± 0.1 nm when freshly synthesized to 10.2 ± 0.1 nm after 14 days and showed a significant statistical difference, albeit no significant change in surface zeta potential was observed (Fig. 8B). Typically, the gradual size increase over time of nanoparticles has been attributed to Ostwald ripening as small less-stable FeNPs merge to become larger more-stable FeNPs.^31^ However, this effect was only in GT-FeNPs but not in pongamol-FeNPs within 14 days. These results suggest that purified pongamol could potentially offer to be more effective stabilizing capping agents than the mixtures of polyphenols in green tea for FeNPs.

Pongamol-FeNPs were also synthesized at varying pongamol concentrations, ranging from 100 µg mL^−1^ to 2000 µg mL^−1^, and all conditions resulted in similar sized particles, as seen in Tables S5 and S6. The resulting FeNPs were consistent at around 10 nm, with surface zeta potential ranging from +19 mV to +30 mV in 1 mM pH 4 sodium acetate buffer. Furthermore, FeNPs have also previously been synthesized with sodium borohydride (NaBH_4_), without any stabilizing agents, resulting in a hydrodynamic diameter of around 10 nm, though these particles exhibited a relatively short stability time of less than 20 seconds.^32^ Our reported synthesis leveraging pongamol provides evidence of stability over a period of 14 days, showcasing that pongamol could act as an effective reducing and stabilizing agent for synthesizing small size FeNPs.

To further assess the colloidal stability of pongamol-FeNPs, a comparison with other plant based green synthesis methods utilizing plant-derived molecules, such as polyphenols, flavonoids, curcumin, and waste biomass extracts are shown in Table 1. Pongamol-FeNPs exhibit excellent stability with no significant change in hydrodynamic diameter over a 14 days period. This is comparable with FeNPs synthesized with tea exact, quercetin, curcumin and Korla fragrant pear peel.^33–39^ However, high stability FeNPs can be synthesized with pure pongamol at relatively low concentration (100 µg mL^−1^), while other plant extracts generally require higher concentrations to facilitate reducing and stabilizing FeNPs. Furthermore, utilizing an isolated compound, like pongamol, as a reducing and stabilizing agent can enhance batch-to-batch consistency, as composition in plant extracts could be sensitive to various factors, including location, season, and species variety, all of which impacts reproducibility and scalability.^40^

**Table 1:** Comparison between pongamol-FeNPs (this work) and other green synthesized FeNPs

Reducing agent	Size (nm)	Morphology	Stability
Pongamol (this work)	∼12	Spherical	>14 days, no visible precipitation for a year
Tea extract^33^	20–80	Spherical	>14 days
Quercetin^34^	10–50	Spherical	14 days
Rutin^35,36^	8–90	Spherical rod shaped	N/A
Curcumin^37,38^	65–406	Spherical	>7 days
*Garcinia mangostana* peel^39^	6–20	Spherical	4 weeks

**Fig. 9:**
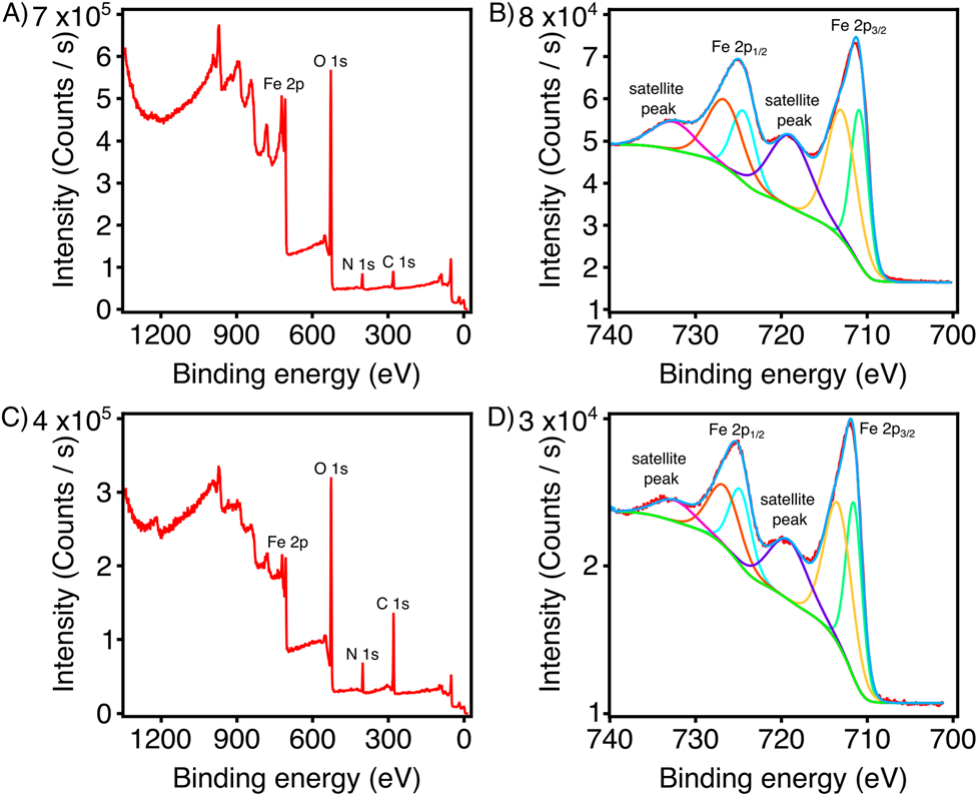
X-ray photoelectron spectra (XPS) of pongamol-FeNPs. (A) Complete XPS survey of freshly prepared FeNPs. (B) Fe 2p XPS spectra of freshly prepared FeNPs. (C) Complete XPS survey of FeNPs after 14 days. (D) Fe 2p XPS spectra of FeNPs after 14 days.

XPS was utilized to help investigate the possible change in oxidation state of Fe in pongamol-FeNPs. According to Fig. 9A and C, oxygen (O), nitrogen (N) and carbon (C) were all observed in pongamol-FeNPs which were freshly prepared and after 14 days. Detected nitrogen confirmed the presence of NO_3_^−^ from ferric nitrate which act as counter ions around the surface of pongamol-FeNPs and could potentially contribute to the formation of elongated structures.^25^ Moreover, the four peaks observed in the Fe 2p spectrum at 732.48 eV, 725.08 eV, 719.78 eV and 711.48 eV suggest pongamol acted as the reducing agent for the formation of FeNPs, which may have formed iron oxide (Fe_2_O_3_) nanoparticles, however, further confirmation may be required using X-ray diffraction (XRD) (Fig. 9B).^41^ The stability of the FeNPs was analyzed by inspecting the Fe 2p spectrum after 14 days. Minimal changes in peak position were observed, namely at the aforementioned peaks at 732.48 eV, 724.98 eV, 719.78 eV and 711.58 eV (Fig. 9D). The consistent XPS results provide support that pongamol (and its functional groups) contribute to stabilizing the oxidation state of FeNPs.

### FeNPs degraded methylene blue effectively as a Fenton reaction catalyst

**Fig. 10:**
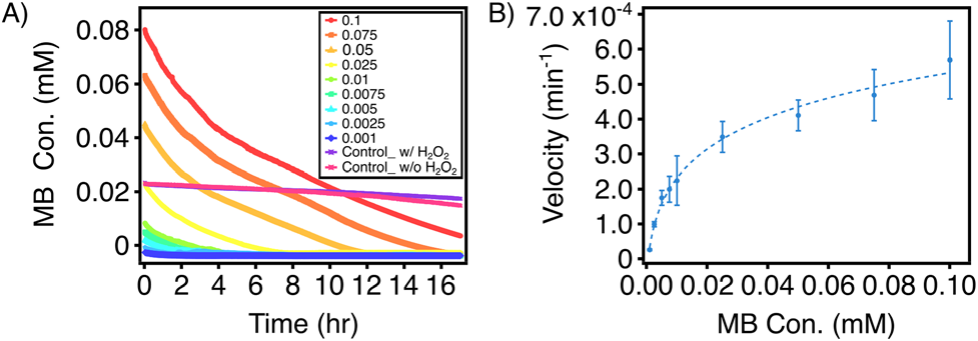
Steady-state kinetic assay of pongamol-FeNPs. (A) Time-dependent degradation of methylene blue (MB) at various initial concentrations (in mM) over a reaction period of 0–17 h. (B) Initial degradation velocity of methylene blue (MB) as a function of initial MB concentration (*n* = 3).

The ability of iron to act as a Fenton reaction catalyst to degrade organic compounds has been widely studied using FeNPs synthesized with various methods.^26^ The mechanism involves the reaction of FeNPs with H_2_O_2_ to generate OH˙ radicals, which attacks the C–S^+^ = C sulfonium bond within the conjugated thiazine ring of methylene blue.^42^ This cleavage results in the loss of double-bond conjugation and opening of the aromatic ring, subsequently leading to decolorization and further degradation of methylene blue. To further evaluate the efficacy and efficiency of the synthesized pongamol-FeNPs as a Fenton catalyst, a steady-state kinetic assay of degradation was performed with methylene blue at various concentrations. As shown in Fig. 10A, no significant concentration decrease was observed for the two control groups, which were absent of any Fenton catalyst. In contrast, a decrease in MB concentration was clearly observed in the presence of FeNPs and the majority of MB were removed within 17 hours. Moreover, the initial degradation velocity was determined by calculating the slope of the concentration values at 0, 1, and 2 min with a standard calibration curve of MB at 664 nm (Fig. S4). The degradation rate was lowest at 0.001 mM MB, increased with higher MB concentrations, and eventually plateaued at 0.1 mM MB (569.34 nM min^−1^) (Fig. 10B).

### Synthesized pongamol-FeNPs promoted *P. defensor* growth at low concentrations

**Fig. 11:**
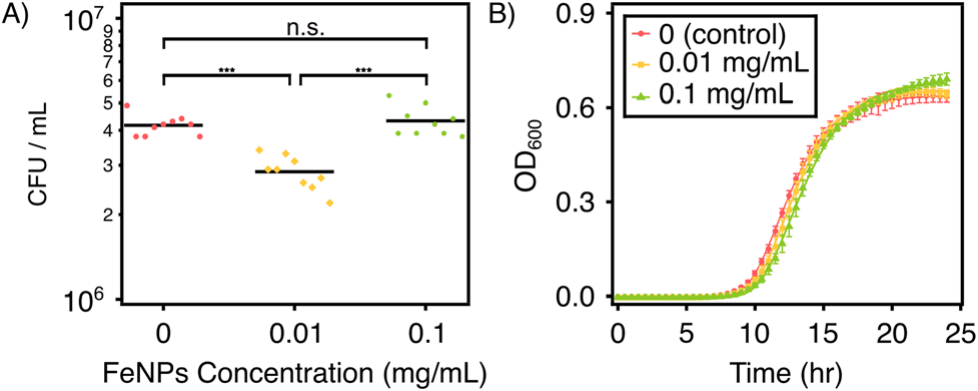
Growth of *P. defensor* under different FeNPs concentrations. (A) On solid medium (King's B agar plates). (B) Gompertz fitting of the growth curve in liquid medium with equation *y* = *a* · exp [−exp(*b* − *ct*)] (King's B medium). Statistical analysis was performed with one-way ANOVA. *P* > 0.05 was considered not significant; ***P < 0.001.

To evaluate the impact of FeNPs on bacterial activity, specifically *P. defensor*, bacterial viability assays were performed with *P. defensor* exposed to various concentrations of FeNPs in both solid and liquid media. According to Fig. 11A, on King's B agar plates, the overall growth of *P. defensor* was not prominent, and the addition of FeNPs resulted in more or less similar CFU counts across treatments. However, there's a significant statistical difference between *P. defensor* after the addition of 0.01 mg mL^−1^ FeNPs and both control and *P. defensor* exposed to 0.1 mg mL^−1^ FeNPs. Furthermore, as shown in Fig. 11B and *P. defensor* growth parameter calculated based on the Gompertz fitting in Table S7, after *P. defensor* were exposed to FeNPs for 24 h, both lag phase and maximum growth increased with increasing pongamol-FeNPs concentrations, while maintaining similar growth rates.^43^ These findings suggested that pongamol-FeNPs at low concentrations could promote *P. defensor* growth. Similar trends have been observed in soil systems, where FeNPs could enhance microbial metabolism and nutrient cycling. He *et al.* stated that iron oxide nanoparticles increased soil microbial metabolic activity and ammonia-oxidizing bacterial abundance which further stimulated carbon and nitrogen cycling and improved soil fertility.^44^ In addition, Guardiola-Márquez *et al.* developed the bio-nanofertilizers composed of γ-Fe_2_O_3_ nanoparticles functionalized with *Pseudomonas* species and *Spirulina platensis* which improved bacterial growth.^45^

## Conclusion

Overall, high purity pongamol (>97%) was obtained by pH-optimized extraction followed by two-solvent recrystallization. This allows for a more accessible and scalable route for isolating pongamol from crude Karanja oil. The purity of the isolated pongamol was found to decrease as pH increased from 5.8 to 6.9. Small and colloidally stable iron nanoparticles were successfully synthesized with pongamol, demonstrating the ability of pongamol to act as both reducing and stabilizing agents at relatively low concentrations, highlighting the importance of exploring alternative compound purification strategies. The application of pongamol-FeNPs to serve as a photocatalyst was effectively demonstrated in degrading methylene blue through a Fenton reaction. Furthermore, pongamol-FeNPs also demonstrated its ability to promote the growth of *P. defensor*. By linking the use of natural plant-derived compounds with environmentally friendly nanoparticle synthesis, our work provides a practical framework for converting other plant-derived flavonoids into functional materials and demonstrate the applications in areas of catalysis and bacteria viability. However, the presence of karanjin in the final product needs further investigation with techniques like quantitative NMR (qNMR) and the extraction method can be further adapted to avoid the usage of hazardous chemicals. Furthermore, additional investigation is required to elucidate the structure and composition of the elongated structures in the synthesized pongamol-FeNPs, the crystallographic phase of the Fe-based nanoparticles, the chemical mechanism underlying the reduction of Fe^3+^ ions by pongamol, and the potential growth-promoting effect of pongamol-FeNPs on *P. defensor*.

## Author contributions

R. C. conceived the study, designed and performed the experiments, and analyzed the data; M. W. assisted with the synthesis of GT-FeNPs and dye degradation, V. P. H. contributed to bacteria viability test, A. G. contributed to TEM imaging of the FeNPs, and F. P. assisted with SEM imaging. N. M. O. A. supervised aspects of the experimental work and provided technical expertise. K. S. K. and R. M. A. S. co-conceived the project and edited the final version of the manuscript. All authors have read and approved the submitted version of the manuscript.

## Conflicts of interest

The authors declare no competing financial interest.

## Supplementary Material

RA-015-D5RA04181C-s001

## Data Availability

Supplementary information (SI): two-solvent recrystallization schematic, HPLC and UV-Vis quantification calibration curve, LCMS target report for Karanja, HPLC peak absorbance at 300 nm and 350 nm, quantitative HPLC peak area data, extraction efficiency of pongamol as a function of pH from HPLC, ^1^H and ^13^C NMR chemical shift assignment, DLS and PALS measurement for FeNPs synthesized with different concentration of pongamol and monitoring for 14 days, calibration curve of methylene blue at 664 nm, and *P. defensor* growth parameter calculated based on the Gompertz fitting. See DOI: https://doi.org/10.1039/d5ra04181c.
