# Supplementary material for: Scalable extraction of pongamol from Pongamia pinnata for synthesis and application of colloidally stable iron nanoparticles
Source: RSC Adv. 2025 Dec 8;15(56):48666–77. doi: 10.1039/d5ra04181c (PMC12684878; doi:10.1039/d5ra04181c)
Supplement: RA-015-D5RA04181C-s001 [file RA-015-D5RA04181C-s001.pdf]

Supplementary Information (SI) for RSC Advances.
This journal is © The Royal Society of Chemistry 2025

Supporting Information

Scalable extraction of pongamol from *Pongamia pinnata* for synthesis and application of colloidally stable iron nanoparticles

Renjie Cui,[a] Mengying Wu,[a] Anna Galang,[b] Franklin Perez,[a] Vanessa Partrige Hing,[a] Nesha May Octavio Andoy,[a] Ruby May Arana Sullan,[a,b*] Kris Sanghyun Kim[a*]

[a]Department of Physical and Environmental Sciences, University of Toronto Scarborough, 1065 Military Trail, Toronto, ON M1C 1A4, Canada

[b]Department of Chemistry, University of Toronto, 80 St. George St., Toronto, ON M5S 3H6, Canada

[*]Corresponding author

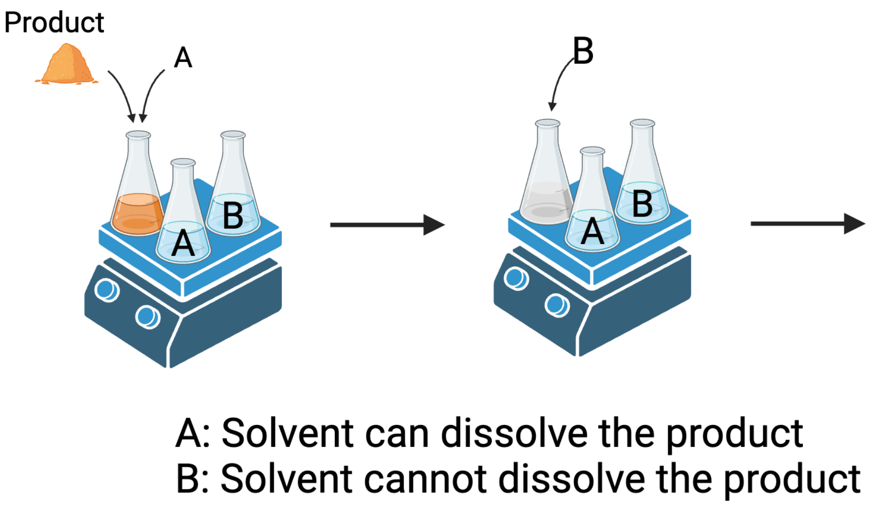

Scheme S1. Schematic depicting two-solvent recrystallization.

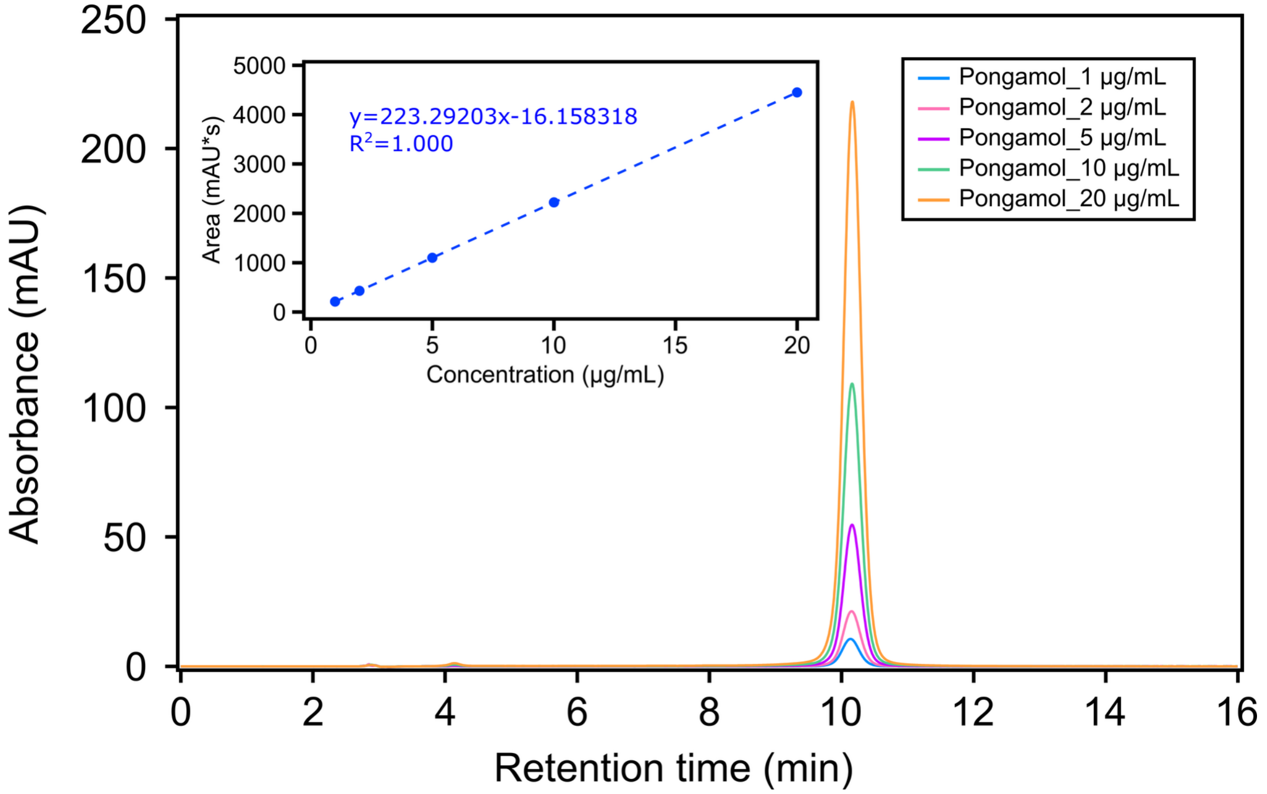

Figure S1. HPLC overlay chromatogram of pongamol at 350 nm. Insert: Calibration curve of pongamol based on a peak at retention time of 10 min with concentrations of 1 μg/mL, 2 μg/mL. 5 μg/mL, 10 μg/mL and 20 μg/mL.

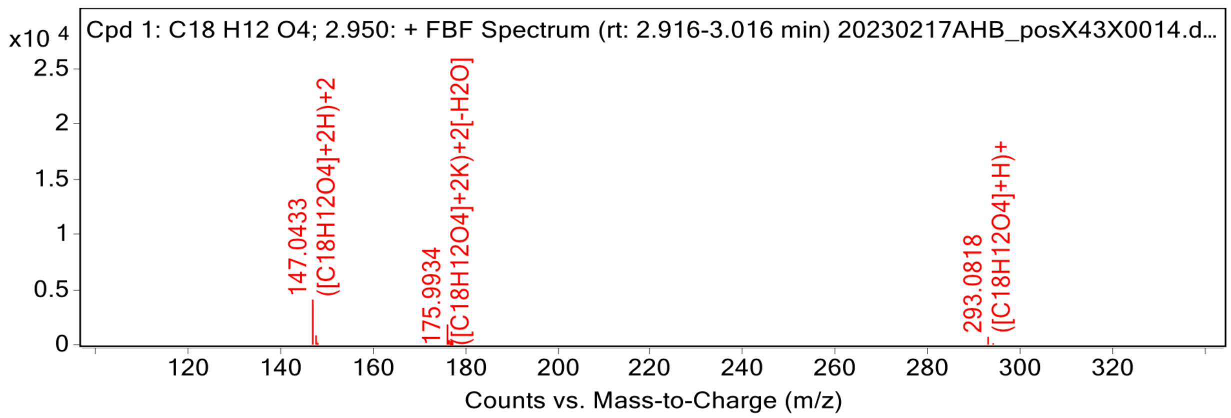

Figure S2. LCMS target compound screening report for Karanja.

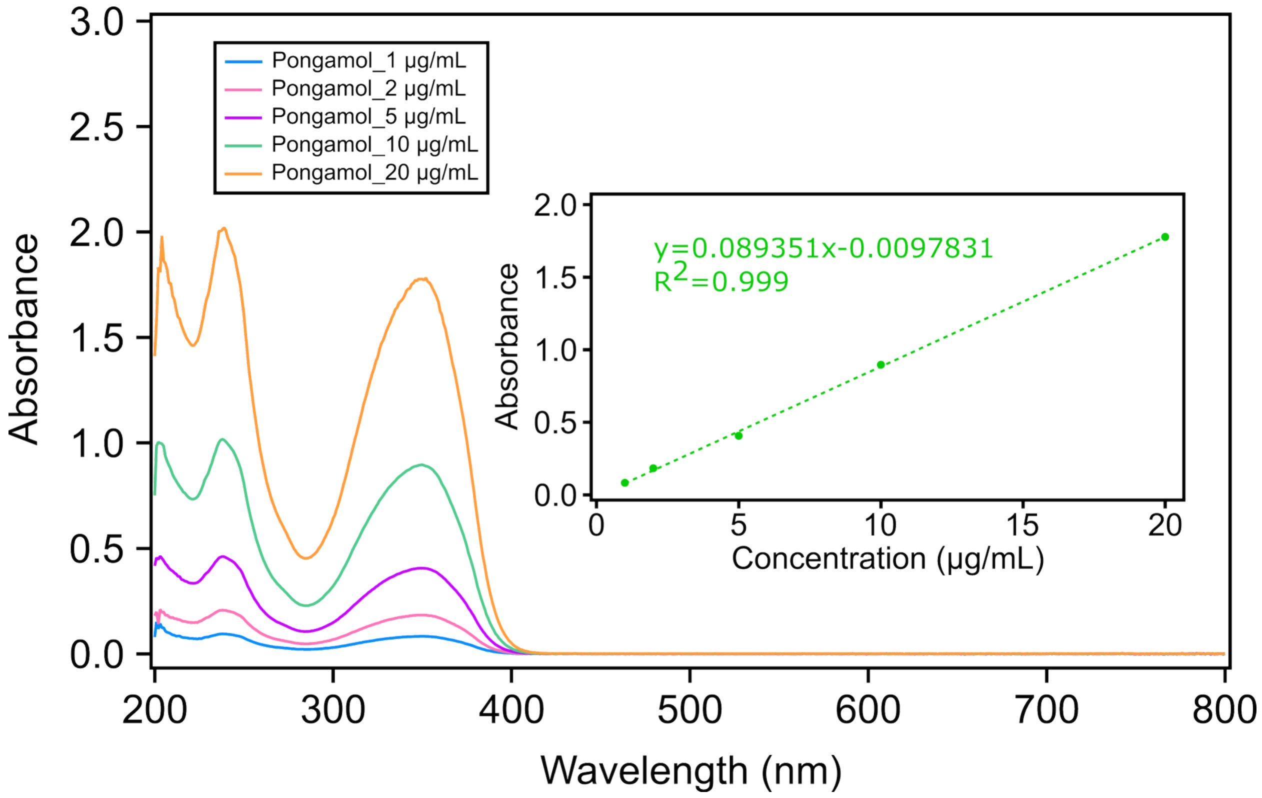

Figure S3. UV-Vis overlay spectrum of pongamol at wavelength from 200 nm to 800 nm. Insert: Calibration curve of pongamol at 350 nm with concentrations of 1 µg/mL, 2 µg/mL. 5 µg/mL, 10 µg/mL and 20 µg/mL.

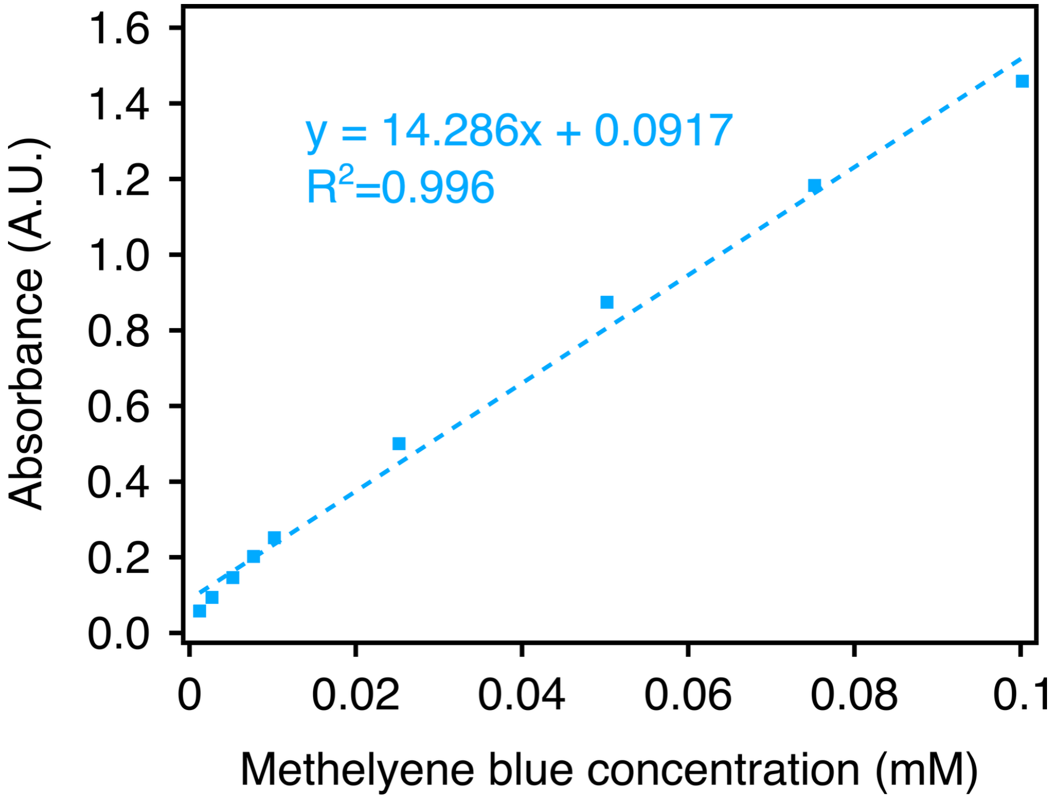

Figure S4. Calibration curve of methylene blue at 664 nm with concentrations of 0.1, 0.075, 0.05, 0.025, 0.01, 0.0075, 0.005, 0.0025, and 0.001 mM.

Table S1. HPLC result of the peaks with retention times at 4 min and 10 min analyzed at wavelengths of 300 nm and 350 nm

Pongamol	Area (mAU * s)			
	Retention time at 4 min		Retention time at 10 min	
	Wavelength 300nm	Wavelength 350nm	Wavelength 300nm	Wavelength 350nm
Extracted	56	18	813	2377
Standard	46	19	792	2280

Table S2. Quantitative HPLC peak area data of pongamol standards (1, 2, 5, 10 and 20 µg/mL) and pongamol extracted at pH 5.8, 6.2 and 6.9 at retention time around 10 min.

Sample	Peak area (at ~10 min) (mAU*s)
Standard 1 µg/mL	210.5
Standard 2 µg/mL	426.9
Standard 5 µg/mL	1098.8
Standard 10 µg/mL	2219.0
Standard 20 µg/mL	4449.1
pH 5.8	2318.0
pH 6.2	2135.7
pH 6.9	1754.2

Table S3. Extraction efficiency of pongamol as a function of pH measured via HPLC.

pH of crude pongamol	Purity (%)
5.8	> 97
6.2	94
6.9	78
Reference acquired from TRC	97.8

Table S4. ^{1}H NMR and ^{13}C NMR chemical shift assignment of pongamol with two structures.

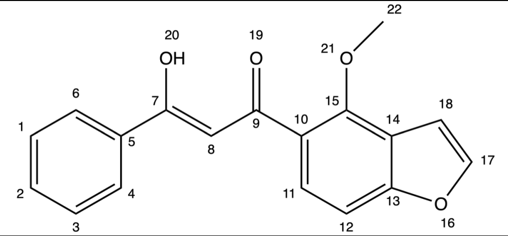

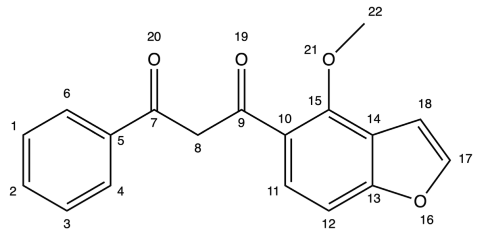

Position	δH (J in Hz)	δC, type	δH (J in Hz)	δC, type
1	7.51, d (8.2, 6.6)	128.65, CH	7.51, d (8.2, 6.6)	128.65, CH
2	7.56, d (7.3)	127.18, CH	7.56, d (7.3)	132.20, CH
3	7.51, d (8.2, 6.6)	128.65, CH	7.51, d (8.2, 6.6)	128.65, CH
4	8.01, d (7.0)	127.18, CH	8.01, d (7.0)	128.75, CH
5		133.37, C		133.37, C
6	8.01, d (7.0)	127.18, CH	8.01, d (7.0)	128.75, CH
7		184.01, C		194.48, C
8	7.19, s	97.96, CH	4.00, s	54.28, CH_2
9		186.14, C		194.97, C
10		122.26, C		122.26, C
11	7.90 / 7.94, d (8.7 / 8.8)	126.56/126.45 CH	7.90 / 7.94, d (8.7 / 8.8)	126.56/126.45 CH
12	7.34, dd (8.7)	107.10/106.87, CH	7.34, dd (8.7)	107.10/106.87, CH
13		159.87/158.72, C		159.87/158.72, C
14		119.64/117.49, C		119.64/117.49, C
15		153.76, C		154.64, C
16				
17	7.66, d (2.3)	144.80/144.95, CH	7.66, d (2.3)	144.80/144.95, CH
18	7.03, d (1.4)	105.24/105.32, CH	7.03, d (1.4)	105.24/105.32, CH
19				
20	16.93, s			
21				
22	4.17, s	61.21, CH_3	4.17, s	61.21, CH_3

Table S5. DLS measurement of FeNP synthesized with pongamol concentrations of 100 µg/mL, 250 µg/mL, 500 µg/mL, 750 µg/mL, 800 µg/mL, 900 µg/mL, 1000 µg/mL and 2000 µg/mL. Measurements were taken on the day when FeNPs were synthesized, after 7 days and 14 days.

Pongamol Concentration (µg/mL)	Fresh (nm)	7 days (nm)	14 days (nm)
100	10.6 ± 0.3	7.9 ± 0.2	10.2 ± 0.2
250	9.4 ± 0.1	10.0 ± 0.3	9.2 ± 0.3
500	9.9 ± 0.4	8.5 ± 0.2	10.1 ± 0.1
750	9.8 ± 0.1	10.0 ± 0.1	10.6 ± 0.2
800	9.7 ± 0.2	9.8 ± 0.2	9.5 ± 0.5
900	9.8 ± 0.2	9.3 ± 0.2	10.1 ± 0.3
1000	9.9 ± 0.1	9.8 ± 0.2	7.9 ± 0.0
2000	9.7 ± 0.1	8.6 ± 0.1	9.8 ± 0.4

Table S6. PALS measurement of FeNP synthesized with pongamol concentrations of 100 µg/mL, 250 µg/mL, 500 µg/mL, 750 µg/mL, 800 µg/mL, 900 µg/mL, 1000 µg/mL and 2000 µg/mL. Measurements were taken on the day when FeNPs were synthesized, after 7 days and 14 days.

Pongamol Concentration (µg/mL)	Fresh (mV)	7 days (mV)	14 days (mV)
100	19 ± 2	20 ± 3	28 ± 7
250	20 ± 8	29 ± 6	26 ± 1
500	22 ± 6	30 ± 4	28 ± 13
750	21 ± 8	51 ± 4	45 ± 1
800	30 ± 4	23 ± 4	41 ± 22
900	23 ± 6	24 ± 4	32 ± 6
1000	23 ± 6	22 ± 2	28 ± 6
2000	21 ± 5	24 ± 3	36 ± 3

Table S7. *P. defensor* growth parameter (lag phase, growth rate and maximum growth) were calculated based on the Gompertz fitting from parameter (a, b and c) from equation $y = a \cdot \exp[-\exp(b - ct)]$

FeNPs concentration (mg/mL)	Lag phase (λ)	Growth rate (μ_m)	Maximum growth (A)
0 (Control)	9.50 ± 0.10	0.108 ± 0.005	0.641 ± 0.024
0.01	9.98 ± 0.01	0.113 ± 0.003	0.655 ± 0.015
0.1	10.31 ± 0.39	0.108 ± 0.010	0.684 ± 0.013
